# Detection and Management of Diabetes during Pregnancy in Low Resource Settings: Insights into Past and Present Clinical Practices

**DOI:** 10.1155/2016/3217098

**Published:** 2016-10-10

**Authors:** Bettina Utz, Alexandre Delamou, Loubna Belaid, Vincent De Brouwere

**Affiliations:** ^1^Department of Public Health, Institute of Tropical Medicine (ITM), Antwerp, Belgium; ^2^Maferinyah Training and Research Centre, Conakry, Guinea; ^3^Centre de Recherche Hospitalier, University of Montreal, Montreal, QC, Canada

## Abstract

*Background.* Timely and adequate treatment is important to limit complications of diabetes affecting pregnancy, but there is a lack of knowledge on how these women are managed in low resource settings.* Objective*. To identify modalities of gestational diabetes detection and management in low and lower middle income countries.* Methods*. We conducted a scoping review of published literature and searched the databases PubMed, Web of Science, Embase, and African Index Medicus. We included all articles published until April 24, 2016, containing information on clinical practices of detection and management of gestational diabetes irrespective of publication date or language.* Results*. We identified 23 articles mainly from Asia and sub-Saharan Africa. The majority of studies were conducted in large tertiary care centers and hospital admission was reported in a third of publications. Ambulatory follow-up was generally done by weekly to fortnightly visits, whereas self-monitoring of blood glucose was not the norm. The cesarean section rate for pregnancies affected by diabetes ranged between 20% and 89%. Referral of newborns to special care units was common.* Conclusion*. The variety of reported provider practices underlines the importance of promoting latest consensus guidelines on GDM screening and management and the dissemination of information regarding their implementation.

## 1. Introduction

The prevalence of diabetes in women of reproductive age is growing in developing countries, where changes in lifestyle are largely accountable for this increase [1]. However, diabetes affecting pregnancy is not yet regarded as a major problem in settings where mothers are dying of more obvious causes such as hemorrhage and hypertensive diseases [2]. Despite a higher risk of affected women to develop direct obstetric complications such as preeclampsia and prolonged labor due to macrosomic babies [3], the attention paid to gestational diabetes (GDM) in low resource countries remains negligible, and not much is yet known about the burden of GDM in these contexts.

Local studies show prevalence rates of GDM between 6% and 14% in East and West Africa [4, 5] and between 13% and 18% in South Asia [6, 7]. There is evidence that treating pregnant women affected by diabetes in pregnancy improves maternal and perinatal outcomes and leads to a fourfold decrease of severe perinatal complications [8, 9]. In mothers who received treatment, excessive growth of a baby and the occurrence of shoulder dystocia and preeclampsia have been reduced [10, 11].

Timely and adequate treatment of affected mothers is key for reducing potential complications particularly when considering the limited timeframe for management from detection until birth. Assuming that one out of ten pregnant women might already be affected by GDM in some low and lower middle income countries, where access to care and resource availability are often major challenges, it is important to understand how and when pregnant women are detected and subsequently managed. Therefore we conducted a review of the published literature to explore documented past and present practices regarding a health problem that will gain increasing attention in the years to come.

## 2. Materials and Methods

### 2.1. Search Strategy

We carried out a scoping review of the literature to broadly map available literature and to identify existing gaps [12]. The electronic databases PubMed, Web of Science, Embase, and African Index Medicus as well as reference lists of identified studies were searched using the following search terms “gestational”, “pregnancy”, “diabetes”, “manage^*∗*^”, “screen^*∗*^”, “developing countr^*∗*^”, “low income countr^*∗*^”, and “lower middle income countr^*∗*^” without methodological, language, or date restrictions. The search strategies were designed to correspond to the specific requirements of each database.

### 2.2. Inclusion and Exclusion Criteria

Articles published until 24 April 2016 that contained any information on clinical practices of detection and management of gestational diabetes/diabetes in pregnancy in low and lower middle income countries as defined by the World Bank [36] were included. We included descriptive and observational studies but excluded intervention studies assessing the prevalence of GDM and publications providing only theoretical recommendations without specific information on observed clinical practices.

### 2.3. Data Extraction and Synthesis

After the removal of duplicates, titles and abstracts were independently screened by two reviewers (BU and AD) regarding their relevance and conformity with the inclusion criteria followed by full text review of selected articles. Any discrepancies could be resolved after discussion between the two reviewers. For reference management we used Endnote version X7 (Thomson Reuters). Data of selected articles was entered into a summary table listing study design, country, year, and study population. Screening and management practices were grouped into the themes screening, hospital admission and inpatient treatment, ambulatory care and follow-up during pregnancy, medication, delivery, newborn, and postpartum care (Table 1).

As management of gestational diabetes was not in all studies distinguishable from the management of preexisting diabetes, we refer to the combination of both as diabetes* during* pregnancy.

## 3. Results

**Figure 1:**
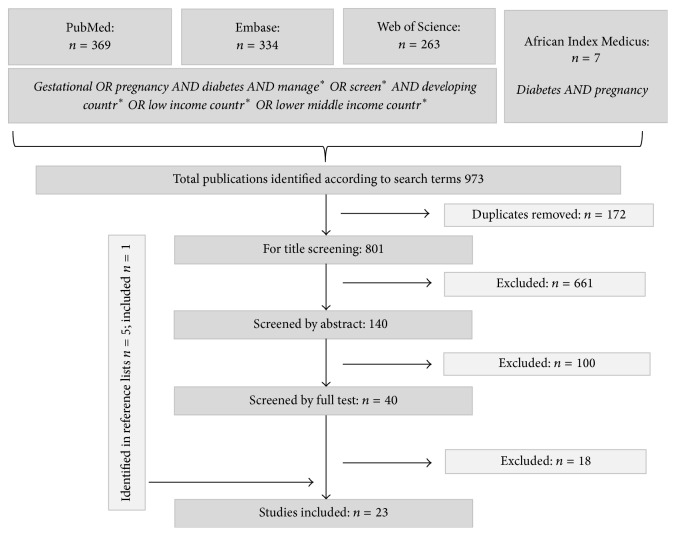
Flowchart of screening process.

Our initial search yielded 973 publications. After the removal of duplicates, 801 papers were retrieved. Through hand searching the bibliographies of screened articles, five additional articles were added. We identified in total 23 papers for inclusion (Figure 1). Of these, eight papers originated from Sub-Saharan Africa with one publication from South Africa [20], three from Nigeria [15, 23, 34], and one from Kenya [14], Tanzania [16], Ivory Coast [18], and Sudan [21]. Two papers were from North Africa with one article from Morocco [24] and one from Libya [17]. There were eleven publications from Asia of which seven papers alone were from India [26–28, 31–33, 35], two publications were from Pakistan [19, 22], one was from Sri Lanka [25], and one was from Vietnam [29]. One paper originated from Fiji [13] and one article included information on six countries in Asia, Africa, and the Caribbean [30].

Aspects of clinical management of pregnant women affected by diabetes were described in 19 articles, and information on screening was provided in 16 publications. The earliest publication dates back to 1977 and the latest article was published in 2015.

### 3.1. Screening

Screening practices varied and available screening tests either used alone or used in combination included in four settings the measurement of random glucose [25, 30, 33, 35] or fasting glucose [24, 26, 30, 33]; postprandial glucose was listed in two studies [24, 25], screening by a glucose challenge test (GCT) was highlighted in five settings [19, 22, 24, 26, 30], and an oral glucose tolerance test (OGTT) was mentioned in 15 out of the 16 studies containing information on screening practices [19–26, 28–31, 33–35]. In one publication, rapid testing of urine for screening was noted [25]. The screening approach was described in 11 studies, with universal testing taking place in five settings [19, 24, 29–31] or as being the preferred approach for the majority of providers in two publications [26, 35]. Selective screening was described in three studies [23, 25, 34] and reported as being preferred by health care providers in one publication [33]. Early selective screening of women with risk factors at their first ANC booking in combination with universal screening after 24 weeks of gestational age was mentioned in two studies [24, 30]. The timing of screening was described in 11 publications and took place between 16 and 24 weeks [35], between 20 and 28 weeks [19], after 24 weeks [25], between 24 and 28 weeks [24, 26], or at 28 weeks [29, 34]. Late screening between 28 and 32 weeks of gestational age was mentioned in one study from Pakistan [22] but was also reported in a publication from Nigeria, where the majority of women (78.7%) were diagnosed with GDM between gestational weeks 28 and 30, whereas 21.3% of women were only detected after 30 weeks of gestation [23]. Screening in every trimester was described in one study from India [31] and in one publication health care providers favored screening at the first ANC visit [33].

### 3.2. Hospital Admission and Inpatient Treatment

Overall, five studies described initial routine hospital admission of pregnant mothers after they had been diagnosed with diabetes [14, 15, 18, 20, 21], whereas in a study from Vietnam, only patients whose glucose levels were not controllable by ambulatory care alone were hospitalized [29]. In a Tanzanian hospital, no specific policy on hospitalization existed for pregnant patients with diabetes and decisions were rather taken on an individual basis to achieve better glucose control and in the presence of obstetric or medical indications [16].

Specific diet advice was described in three studies and patients were instructed to follow a diet restricted in carbohydrates [14] or in calories [15, 20]. Information on glucose monitoring during hospitalization was reported in five papers and glucose values of hospitalized patients were measured regularly from two to six times within 24 hours [14, 15, 20, 21], either on a daily basis [20] or once or twice a week [14, 15, 18, 21]. In a study from Ivory Coast, blood and urine tests, vaginal swabs, ophthalmological checks, and ultrasound investigations were performed during hospitalization [18]. Patient education included diet counselling in two papers [18, 20] and the initiation to self-monitoring of blood glucose (SMBG) in one publication [20]. In five studies, self-monitoring was reported as not being performed [16, 17, 21, 23, 28]. Timing of discharge was specified in three studies and women were discharged after two weeks [18] or after glucose values were sufficiently controlled [14, 21].

Admission or readmission in gestational weeks 32 to 34 was identified in four studies [13–15, 21]. In one publication [17], only high risk pregnant mothers with diabetes during pregnancy were admitted in week 34/35, three to four weeks earlier than women with a normal or low risk, whereas in another publication patients were readmitted only around term [18]. Regarding the management of readmitted patients, reported practices ranged from bed rest [13], surveillance of caloric intake [13], blood glucose measurements [13, 18, 21], control of fetal wellbeing [18], and daily measurements of urine and blood pressure to weekly measurements of weight and uterine height [14]. In two publications dating back more than 30 years, amniotic fluid was examined once a week from 36 weeks onwards to assess fetal pulmonary maturity [13, 14] with corticosteroids being administered a week before the planned delivery [13].

### 3.3. Follow-Up during Pregnancy

Outpatient follow-up intervals of women affected by diabetes during pregnancy were described in eight papers and ranged from weekly [18, 20, 29], weekly to fortnightly [14, 17, 23], every two to three weeks, unless the diabetes was poorly controlled [16], to monthly controls [19] with reported shorter intervals in the third trimester indicated in two publications [19, 23]. Health care provider surveys from India revealed that providers preferred to control twice monthly [27, 33] often in combination with self-monitoring [27]. In one publication from India it was considered sufficient to present the results of glucose tests performed in private laboratories every two weeks [28]. Outpatient care included nutritional advice [18, 20, 28, 29, 31], control of glycaemia [14, 16–18, 23, 28, 29], and monitoring of urine [14, 16, 23], weight [14, 16], blood pressure [14], and fetal growth [14, 17, 22, 23]. Six articles highlighted the multidisciplinary character of the care teams [17, 20–23, 34]. According to a provider survey from India, patients with GDM were usually referred to a specialist [27].

### 3.4. Medication

According to 13 articles, insulin was the treatment of choice if diet alone was not sufficient to control hyperglycemia [13–18, 20, 21, 23, 28, 29, 31, 33]. Insulin was started as early as three days after diet modification [20] or after two weeks of diet [28] and was administered between once and three times daily [14, 15, 17, 28]. Regarding diabetes control, poor control was reported for 6% of diabetic mothers in a setting in South Africa [20], for 20% in a study from Sudan [21], for 40% of patients in a study from Nigeria [23], and for 58% of pregnant diabetics in a publication from Pakistan [19].

### 3.5. Delivery, Newborn, and Postpartum Care

Specific information regarding the mode of delivery, intrapartum and newborn care, and maternal follow-up postpartum were mentioned in 14 studies [13–17, 20–23, 27, 31, 32, 34, 35]. Induction in week 37/38 was the preferred mode of delivering high risk pregnant patients with diabetes whereas medium and low risk patients were allowed to deliver vaginally at term in one publication [17]. In four papers, labor was induced at a gestational age of 38 to 39 weeks [21–23, 31] corresponding to reported health care provider practices from India where the majority of obstetricians delivered women with GDM up to a gestational age of 38 weeks [27]. In a study from Pakistan, women who delivered vaginally were routinely assisted by vacuum or forceps in the second stage of labor [22]. In a publication from Tanzania [16], labor was not routinely induced and vaginal delivery attempted. This corresponds to findings from South Africa, where pregnancies were allowed to proceed to term given that glycemic control was good and no other obstetric complications were present [20]. In two studies, delivery took place electively after pulmonary maturity had been ascertained [13, 14], either vaginally or by caesarean section in case of obstetric indications [13]. Caesarean section rates described in 14 studies ranged between 20% and 89% with a median rate of 42%. Glucose control in labor was mentioned in two articles reporting two-hourly monitoring of parturients [21, 22]. Regarding the care of the newborns of diabetic mothers, a study from Kenya mentioned the presence of pediatricians at each delivery and observation of the newborns for several days [14]. Referral of newborns was reported in five studies. In one publication all newborns were admitted to the special care unit [15] and in two publications nearly 50% of newborns were referred to special care [22, 34]. In a hospital setting in India, all newborns of mothers receiving antidiabetic medication were admitted to special nursing care where they were regularly fed and referred back after achieving normal glucose levels [32]. Results of a provider practice survey in India revealed that one-third of clinicians refer every second newborn and 57% of providers refer one in 10 babies [27].

Retesting of women six weeks postpartum for a repeat 75 g OGTT was mentioned in one article [23] and in two papers between 54% and 93% of clinicians stated they would recommend a postpartum test [27, 35].

### 3.6. Limitations

Our descriptive approach enabled us to shed some light on the diversity of screening and management procedures regarding diabetes during pregnancy, although the identified studies reflect only individual practices in specific hospitals or reported provider practices. National guidelines, where available, would have provided a more comprehensive picture of the national situation but as their availability alone does not necessarily mirror performed practice, we intentionally choose to identify only observed or reported clinical practices. As some of the publications date back almost 40 years, practices have changed over time and may not reflect the actual situation anymore. A specific focus on gestational diabetes practices was not always possible as some authors did not distinguish between preexisting diabetes and GDM when reporting on management practices.

## 4. Discussion

Our review revealed a limited number of publications describing detection and management practices of gestational diabetes in low resource settings. The majority of studies were conducted in large tertiary hospitals and although this may be partly due to publication bias with providers from referral centers more likely to publish, the findings indicate that the majority of patients with diagnosed diabetes during pregnancy are usually referred and treated through higher level services, where specialists and multidisciplinary teams are available.

Screening for GDM in developing countries is often not performed routinely and where applied, screening approaches are not uniform. Modes of screening vary not only between countries but also within countries, even within the same service [24], and highlight the importance of training health care providers on uniform practice standards that are applicable to low resource settings.

Therapeutic options for pregnant women diagnosed with GDM include nutritional therapy and antidiabetic medication. Although nutritional therapy has been highlighted in most of the publications as the foundation of treatment, overreliance on adequate nutrition coupled with a potential anxiety to administer insulin by health care providers based on a fear of hypoglycemia [37] might be one of the reasons for poorly controlled diabetes during pregnancy. Furthermore, information on nutrition may be misleading if recommendations are not adapted to local dietary habits and food availability [29].

In most publications, insulin was the drug of choice for pregnant women whose diabetes was not controlled by diet alone. Availability and storage issues of insulin are particular barriers to adequate management in low resource settings [38] and underline the need for a safe oral alternative. Recent international consensus guidelines of the International Federation of Gynecology and Obstetrics (FIGO) include Metformin as first-line treatment [39] which has proven to be a safe oral alternative to insulin [40] and more feasible to use in settings where regular monitoring of blood glucose cannot be assured.

Our results revealed that in several settings patients were initially admitted for nutritional education, close glucose monitoring, and initiation of treatment, although it seems that over time there was a trend to ambulatory management. In some of the earlier studies, bed rest was advised for pregnant women with diabetes, a practice for which current evidence is missing [41]. Hospitalization for treatment initiation and improved monitoring might be considered for settings where regular access to health services is not secured, although inpatient treatment might have substantial financial implications for affected women and their families [42].

Accessibility to regular follow-up as well as accessibility to self-testing of blood glucose for women with GDM are challenges that need to be addressed. In the majority of studies, ambulatory glucose controls were performed weekly to fortnightly and ranged from measuring urine glucose and random or fasting blood glucose to postprandial glucose checks. In several settings, self-monitoring of glucose was not considered a feasible option often due to difficulties for patients to purchase equipment. Other factors such as not feeling comfortable to self-monitor or to interpret blood sugar results at home made women return to the hospital for monitoring [29]. Contextualized local solutions to ensure regular monitoring in proximity to the women need to be developed, offering an alternative to self-monitoring and might include support through peers or care providers based within the communities.

## 5. Conclusion

In various settings, certain aspects of the above described management of GDM might still prevail and have possibly been adapted to local circumstances. However, the paucity of published information on clinical practices coupled with a lack of uniformity in the management of gestational diabetes requires a focus on the promotion of universal guidelines on GDM screening and management that are applicable to low resource settings. With the recent publication of the FIGO consensus recommendations grouped according to income setting [39], an important step has been taken. Information about their implementation and examples of best practice, particularly from low income settings, need to be disseminated.

## Figures and Tables

**Table 1:** Screening and management practices in identified publications.

Number	Author (ref.)	Year	Country	Setting	Design	Population	Screening	(Re)admission	Inpatient care	Follow-up	Outpatient care	Delivery	Newborn care	Postpartum
1	Sutton [13]	1977	Fiji	Referral hospital	Retrospective observational	21 pregnant diabetic women		32 weeks	Bed rest, diet, glycaemia 2x weekly. Amniotic fluid 1x weekly at ≥36 weeks; steroids 1 week prenatal			Planned delivery at 38 weeks: vaginal delivery but CS if complications or labor >18 hours; observed CS rate 57%		

2	Fraser [14]	1982	Kenya	National hospital	Retrospective observational	51 pregnant diabetic women	50 g OGTT	After first visit (<32 weeks); Readmission 32 weeks	Initial stay: diet, glycaemia 3x/day 1-2/week; readmission: urine & blood pressure daily; weight & uterine height weekly; ≥37 weeks: amniotic fluid weekly	Weekly or fortnightly	Glucose and urine, blood pressure, weight, and abdominal examination	Induction; CS if no delivery within 12 hrs or if indication; observed CS rate 31% (half of them elective)	Pediatricians at delivery; observation of newborn for several days; early feeding	

3	Otolorin et al. [15]	1985	Nigeria	University hospital	Retrospective observational	48 pregnant women diagnosed with diabetes		Initial admission first trimester/after booking; readmission between 32–34 weeks	Diet with 2000–2500 cal; twice weekly 4-point profile			Mode of delivery depending on several factors (e.g. age, diabetes control). Observed CS rate of 41%; 70% of patients delivered before 38 weeks	All newborn admitted to special newborn care unit and reviewed by pediatrician	

4	Lutale et al. [16]	1991	Tanzania	University hospital	Prospective observational	47 pregnant diabetic women		No specific policy; decision on individual basis		Every 2-3 weeks; weekly if poorly controlled	Glucose and urine test, weight; no SMBG	Vaginal delivery; labor not routinely induced in uncomplicated pregnancies; induction only if shake test positive; observed CS rate 30%		

5	Kadiki et al. [17]	1993	Libya	Urban diabetes clinic	Retrospective observational	988 pregnant diabetic patients		High risk patients admitted at 34-35 weeks; all others in week 37-38		Fortnightly until 24 weeks, weekly thereafter	Fasting and postprandial plasma glucose; no SMBG; ultrasound to monitor fetal growth	Vaginal delivery: induction of high risk patients in week 37-38; all others allowed to proceed to term; observed CS rate 36%		

6	Djanhan et al. [18]	1995	Ivory Coast	University hospital	Prospective observational	109 pregnant women diagnosed with diabetes		Initially for 2 weeks; readmitted around term	Initial stay: glycaemia, blood, and urine tests, vaginal swab, ophthalmological check, ultrasound, and diet counselling. Readmission: FHB daily, every 2nd day fasting glucose	Weekly; obstetrician monthly		Observation: 95% delivered at term		

7	Akhter et al. [19]	1996	Pakistan	University hospital	Retrospective observational	267 diabetic pregnancies	Universal screening: 50 g GCT weeks 20–28; women with RF/abnormal GCT: 75 g OGTT			Monthly; fortnightly in third trimester		Observed CS rate: 26%		

8	Daponte et al. [20]	1999	South Africa	University hospital	Retrospective observational	142 pregnant women with diabetes	100 g OGTT	Admission for education on glucose monitoring and diet	6-point glucose profile daily, diet counselling (1800–2000 cal) & SMBG initiation	Weekly by multidisciplinary team		Women allowed to proceed to term if good glycemic control and no other obstetric complications; observed CS rate 49%; mean gestational age at delivery 38 weeks		

9	Mirghani and Saeed [21]	2000	Sudan	Teaching hospital	Prospective observational	74 pregnant women with diabetes	75 g OGTT	Initial admission; readmission weeks 34–36	Initial admission: urine 6 hourly and glycaemia 2x/week; readmission: FBG 2x/weekly	Fortnightly ANC: FBG (no SMBG possible)		Delivery 38 weeks (induction or CS if not delivered within 12 hours), during labor glycosuria & glycaemia, prophylactic antibiotics. Observed CS rate 65%	Breast feeding 30 min after delivery and 4–6 hours after CS. Pediatrician present; newborn blood sugar 2 hours after birth	

10	Randhawa et al. [22]	2003	Pakistan	Teaching hospital	Retrospective observational	50 women with GDM and diabetes in pregnancy	GCT followed by OGTT in weeks 28–32				Initial advice on diet and exercise; regular ultrasound for fetal growth, FHR 2x/weekly, biophysical profile weekly in high risk cases after 32 weeks	Induction at 38 weeks; CS if >4000 g; in labor FHB and 2-hourly glycaemia; 2nd stage assisted; prophylactic antibiotics. Observed CS rate 50%	No specific information provided, but 48% of newborns admitted to neonatal ward	

11	Ozumba et al. [23]	2004	Nigeria	University hospital	Retrospective observational	207 pregnant women diagnosed with diabetes	Selective screening; 75 g OGTT; fasting glucose of women with known diabetes			Fortnightly until 32 weeks, weekly thereafter. Follow-up in ANC and by physician in diabetes clinic	Fasting and postprandial glucose, ultrasound, blood grouping, and rhesus factor, hemoglobin, and urine. No SMBG (only if women can purchase glucometer)	Induction at 38 weeks. Vaginal delivery in uncomplicated and well-controlled cases; induced if poorly controlled or complications; observed CS rate in GDM patients 20%		Women invited for repeat 75 g OGTT 6 weeks postpartum

12	Bouhsain et al. [24]	2009	Morocco	Teaching hospital	Retrospective observational	702 pregnant women consulting the gynecology department	If RF: screening at first ANC; universal screening at 24–28 weeks; screening with FBG alone or in combination with postprandial glycaemia or 50 g GCT followed by 100 g OGTT in case of GCT positivity							

13	Dahana-yaka et al. [25]	2011	Sri Lanka	District facilities	Cross-sectional descriptive	223 pregnant women attending antenatal clinics	Selective screening at >24 weeks: 30.2% women with RF screened. 98% use urine dipstick, 27% postprandial glycaemia, 11% FBG or RBG, and 3% 75 g OGTT							

14	Divakar & Manyonda [26]	2011	India	NA	Cross-sectional survey	584 specialists OBGY	Universal screening by 82% respondents; 65.5% test at first visit, 97.6% in weeks 24–28; as test 50g GCT done by 39.3%; 75 g OGTT by 26.2%; 14.3% test FBG.							

15	Divakar & Manyonda [27]	2012	India	NA	Cross-sectional survey	584 specialists OBGY				Fortnightly glucose; 47.6% respondents advise daily home monitoring combined with follow-up visits 2x/month	69.1% of clinicians refer women with GDM to specialists	64.3% of obstetricians deliver women with GDM ≤ 38 weeks; 35.7% await spontaneous labor but 54.8% wait no longer than 39 weeks	57.1% of clinicians refer 10% and 33.3% refer 50% of newborns of mothers with GDM to NICU	93% of doctors advise testing 6 weeks postpartum: 56% advise random glucose tests

16	Maiti et al. [28]	2012	India	Urban hospital	Prospective observational	50 women with GDM	75 g OGTT			Women or relatives present results of fortnightly glucose test at clinic every 2 weeks	Nutritional advice; 3-point profile fortnightly at laboratories close to patient's home (no SMBG)	Observed CS rate (GDM): 84%; 82% delivered at term		

17	Hirst et al. [29]	2012	Vietnam	Referral hospital	Qualitative study on perceptions & experiences of pregnant women with GDM management	4 FGD with 34 women having gestational diabetes	Universal screening; 75 g OGTT in week 28	Admission of noncontrollable cases	Glucose monitoring up to 6x daily	Weekly follow-up; glucose checks once or twice weekly at OPD if no SMBG	Women with GDM referred to high risk antenatal clinic: physician provides advice on nutrition. Glucose-surveillance recommended by SMBG or 1-2x/week at OPD			

18	Nielsen et al. [30]	2012	Cameroon, China, Cuba, India, Kenya, and Sudan		Retrospective descriptive; review of screening practices of 9 GDM projects and qualitative assessment of barriers		Universal screening in 78% of 9 GDM projects by random glucose testing (Sudan), fasting glucose followed by OGTT (Cuba, Cameroon, and China); GCT followed by OGTT (Karnataka, India); or OGTT alone (Kenya & 2 states of India)							

19	Rajagopalan et al. [31]	2013	India	Private hospital	Retrospective observational	Screening practices of 753 women booked in ANC; 105 with GDM	Universal screening; 2010–2012: single step at 24 weeks; 2013: screening in each trimester at booking, 26 and 34 weeks				After diagnosis advise on diet, exercise (and medication)	Induction of labor between 38 and 39 weeks; observed CS rate 38%		

20	Thomas et al. [32]	2013	India	University hospital	Prospective observational	281 women with GDM requiring medication			Glycaemia 3–7 days after diet initiation			Observed CS rate: 43%; mean gestational age at delivery 37.5 weeks	Referral to nursing care: hourly feeding first 6 hours, then 2-hourly; glucose test after 1, 3, 5, 9, and 12 hours; if hypoglycemia iv dextrose	

21	Gupta et al. [33]	2014	India	NA	Cross-sectional survey	134 health care providers (56 OBGY, 78 physicians)	59.7% of providers screen selectively based on RF and 30% screen universally; 88.8% respondents screen at first ANC visit: 77.6% of professionals by FBG, 18.6% by RBG, and 3.8% use 75 g OGTT			62.7% providers advise glucose test once every 2 weeks, 28.4% weekly				

22	John et al. [34]	2015	Nigeria	University hospital	Retrospective observational	122 pregnant women with diabetes and 101 with GDM	Selective screening at booking with 75 g OGTT; repeated at 28 weeks					Mode of delivery assessed on individual basis depending on glycemic control; observed CS rate 89%	49% of newborns admitted to NICU	

23	Babu et al. [35]	2015	India	70 public health facilities	Cross-sectional survey	50 doctors	Universal screening by 82% doctors: 52% in weeks 16–24. Screening by RBG done by 46% of respondents; GDM diagnosis with 75 g OGTT by 96% respondents							54% doctors test sugar postpartum and 36% use FBG; 80% counsel on diet; 82% on exercise; 96% advise follow-up of glycaemia
